# A NGS-Targeted Autism/ID Panel Reveals Compound Heterozygous GNB5 Variants in a Novel Patient

**DOI:** 10.3389/fgene.2018.00626

**Published:** 2018-12-12

**Authors:** Natascia Malerba, Shelley Towner, Katherine Keating, Gabriella Maria Squeo, William Wilson, Giuseppe Merla

**Affiliations:** ^1^Division of Medical Genetics, IRCCS Casa Sollievo della Sofferenza, San Giovanni Rotondo, Italy; ^2^Division of Medical Genetics, University of Virginia, Charlottesville, VA, United States

**Keywords:** GNB5, IDDCA, LADCI, cognitive impairment, cardiac arrhythmia

## Abstract

Homozygous and compound heterozygous pathogenic variants in *GNB5* have been recently associated with a spectrum of clinical presentations varying from a severe multisystem form of the disorder including intellectual disability, early infantile developmental and epileptic encephalopathy, retinal abnormalities and cardiac arrhythmias (IDDCA) to a milder form with language delay, attention-deficit/hyperactivity disorder, cognitive impairment, with or without cardiac arrhythmia (LADCI). Approximately twenty patients have been described so far; here we report a novel case of a 2.5-year-old female who is a compound heterozygote for a frameshift and a missense variant in the *GNB5* gene. Her clinical presentation is consistent with a moderate phenotype, corroborating the direct correlation between the type and pathogenic mechanism of the *GNB5* genetic variant and the severity of related phenotype.

## Introduction

G protein coupled receptors (GPCR), by activating heterotrimeric guanine nucleotide-binding proteins (G-proteins), control an array of cellular functions (Smrcka, 2008). G-proteins are composed of α, β and γ subunits. In the inactive state, the GDP-bound Gα subunit is associated to the Gβγ heterodimer. Ligand binding of a GPCR acts as guanine-nucleotide exchange factors (GEFs) that activate trimeric G-proteins, leading the exchange of GDP in GTP on the Gα subunit, its dissociation from Gβγ dimer, and thereby enabling both Gα and Gβγ subunits to modulate a wide range of downstream signaling (Gilman, 1987). GPCR cascade is ended by the regulator of G-protein signaling (RGS) proteins, which accelerate the GTP hydrolysis on the Gα subunits, thereby promoting their inactivation. In the nervous system, the control of GPCR signaling is achieved by the members of the R7 subfamily of RGS proteins (R7-RGS). A hallmark of R7-RGS protein organization is their association with Gβ5, encoded by GNB5, a divergent member of the beta subunits of heterotrimeric G proteins (Cabrera et al., 1998; Makino et al., 1999).

In humans, variants in each of the five distinct G proteins β-subunits (*GNB1*-*5*) cause developmental delays and/or heart rhythm disorders. Germline *GNB1* variants cause severe neurodevelopmental disability, hypotonia, and seizures (Petrovski et al., 2016); *GNB2* variants cause sinus node and atrioventricular conduction dysfunction (Stallmeyer et al., 2017); *GNB3* bi-allelic loss-of-function variants cause congenital stationary night blindness (Vincent et al., 2016), recessive retinopathy (Arno et al., 2016), and a reduced cone sensitivity with bradycardia(Tummala et al., 2006; Ye et al., 2014); finally, *GNB4* variants cause a dominant form of Charcot-Marie-Tooth disease (Soong et al., 2013).

Recently, others and we identified homozygous or compound heterozygous variants in the *GNB*5 gene that are associated with either IDDCA (MIM#617173) or LADCI (MIM#617182), in which the severity of the phenotype correlates with the type of the genetic variant (Lodder et al., 2016; Shamseldin et al., 2016). Specifically, homozygous carriers of the most frequent missense variant p.(Ser81Leu) are characterized by mild intellectual disability in combination with language delay, attention-deficit/hyperactivity disorder, with or without cardiac arrhythmia (LADCI) (Shamseldin et al., 2016). In contrast, homozygous carriers of null alleles showed the severe IDDCA neurological phenotype including epileptic seizures, intellectual disability, retinal abnormalities, hypotonia, and sick sinus syndrome (Lodder et al., 2016). Consistent with the manifestations of IDDCA patients, phenotypic characterization of *Gnb5* loss in mice and our zebrafish *gnb5* knockout showed, respectively, abnormalities of neuronal development, including hyperactivity and an altered motor capacity, impaired ocular response, and cardiac sinus node dysfunction (Chen et al., 2003; Wang et al., 2011; Zhang et al., 2011; Xie et al., 2012; Lodder et al., 2016), corroborating the importance of *GNB5* for neuronal signaling and parasympathetic regulation of heart rate as well as vision and motor function.

After the first cases described (Lodder et al., 2016; Shamseldin et al., 2016) additional cases have been reported (Turkdogan et al., 2017; Vernon et al., 2018). Here we report a new patient with compound heterozygosity for the p.(Asp74Glufs^∗^52) frameshift variant and the p.(Ser81Leu) missense variant in *GNB5*. Her clinical features are consistent with a moderate phenotype, suggesting the direct correlation between the type and likely the mechanism of the *GNB5* genetic variant and the severity of phenotype.

## Background

### Case Report

**FIGURE 1:**
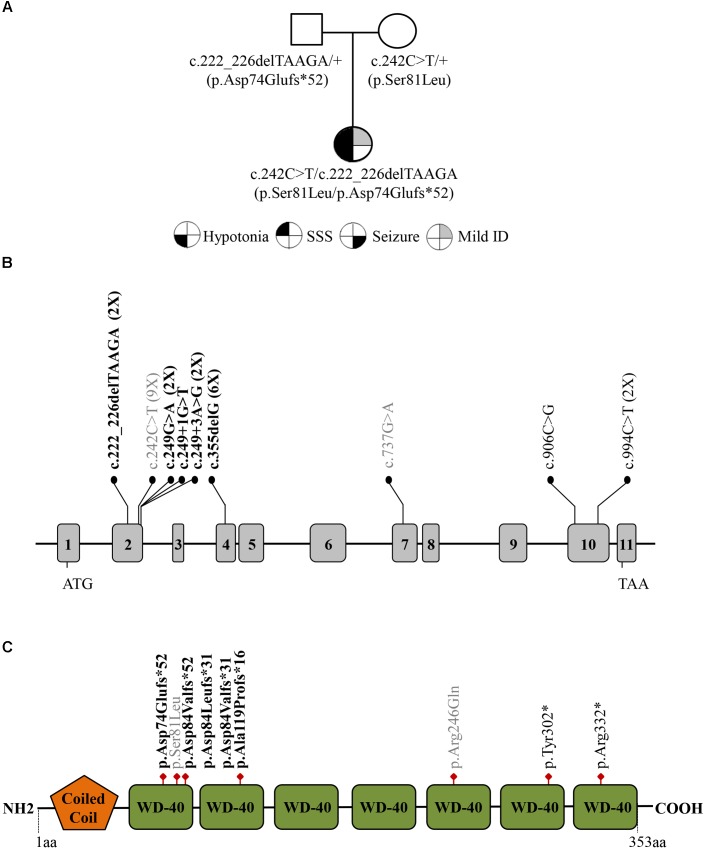
**(A)** Family pedigree investigated in this study. Filled symbols represent: hypotonia (bottom left quarter), severe sick sinus syndrome (SSS; top left quarter), seizure (bottom right quarter), and intellectual disability (ID; top right quarter). **(B)** Distribution of the GNB5 variants (NM_006578) across the gene with exons indicated by gray boxes. The frameshift variants are designated in bold, missense variants are in gray, and nonsense variants are in black. The number of patients with each variant is indicated in parentheses. **(C)** Schematic representation of the GNB5 protein with Coiled coil and WD-40 repeats domains.

We describe a 2.5-year-old female proband, born from non-consanguineous and healthy parents of Caucasian ancestry (Figure 1A). The child was the product of a 35-week gestation pregnancy that was complicated by fetal bradycardia and intrauterine growth retardation that prompted an emergency C-section. The family history was negative for developmental delay, sick sinus syndrome, and epilepsy, with the exception of a maternal uncle and a paternal first-cousin, both suspected to have autism spectrum disorder.

At birth she required positive-pressure ventilation for 3 min. Apgar scores at 1 and 5 min were 1 and 7, respectively. Birth weight was 1698 g (<1st percentile). She was hospitalized for 3 weeks in the neonatal intensive care unit for prematurity and intrauterine growth restriction being less than 2000 grams. Due to her feeding difficulties, she was fed by nasogastric tube. After 20 days she was discharged home on full oral feeds. At 8 months of age she was noted to have plagiocephaly associated with torticollis, solved with molding helmet therapy. The typical developmental milestones were not met at 1 year of age. When she was 13 months old her gross motor skills were noted to be at the 10-month level, her fine motor skills and receptive language skills were at the 9-month level, and her expressive language skills were at the 5-month level, indicating delays of 3 to 8 months. At 15 months, her developmental quotient, calculated by Capute scores (Accardo and Capute, 2005) was between 50 and 55; therefore, her psychomotor delay was assessed as ranging between mild to moderate.

She had strabismus, which was surgically corrected at 16 months. At the same age, she also had implantation of bilateral tympanostomy tubes. Following intensive therapies, her developmental skills have improved. She began walking independently at 18 months. Additionally, she has hypotonia, a wide-based gait, and poor balance. She was described as falling more frequently than expected. However, her communication, particularly expressive language skills, represents the biggest challenge. Currently, her spoken vocabulary consists of about 12 words, and she can also use some sign language. She is social, curious, and interactive and has a high activity level and short attention span compared to peers.

Following identification of her *GNB5* variants and the reported clinical spectrum of IDDCA which includes early-onset sick sinus syndrome and epileptic encephalopathy among other cardinal manifestations, the patient was referred to cardiology and neurology.

An electrocardiogram (ECG) showed sinus bradycardia with heart rate of ∼50 at the age of 18 months. A 24-h Holter monitor showed evidence of sinus node dysfunction, with over 3,000 sudden rate drops and pauses that exceeded 3 s. A pacemaker was placed at 2 years of age. Her frequent falls and poor balance improved after pacemaker placement, raising the possibility that those falls were related to sinus pauses. The neurologic evaluation was normal; no evidence of epileptic encephalopathy was detected. Due to lack of relevant clinical findings, an EEG was not performed.

## Materials and Methods

As no potentially pathogenic genomic structural abnormalities were identified by SNP array-CGH of the subject, genomic DNA from the proband and her parents were analyzed by next-generation sequencing using the Autism/ID Xpanded Panel (GeneDx^1^) on an Illumina sequencing System. Reads were aligned to human genome build GRCh37/UCSC hg19, and analyzed for sequence variants in targeted genes using a GeneDx custom-developed analysis tool (Xome Analyzer). Sequence and copy number alterations were reported according to the Human Genome Variation Society (HGVS) and International System for Human Cytogenetic Nomenclature (ISCN) guidelines, respectively.

The patient’s biospecimens (DNA and skin fibroblast cell lines) are stored at Genomic Disorder Biobank, member of the Telethon Network of Genetic Biobanks (Filocamo et al., 2013; Mora et al., 2014). This study was carried out in accordance with the recommendations of the University of Virginia Institutional Review Board for Health Sciences Research. The subject gave written informed consent in accordance with the Declaration of Helsinki. Written informed consent was obtained from the parent of the patient for the publication of this case.

### Molecular Results

The proband’s results were compound heterozygous for a maternally inherited missense substitution [c.242C > T; p.(Ser81Leu)], and a paternally inherited frameshift variant [c.222_226delTAAGA; p.(Asp74Glufs^∗^52)] in the *GNB5* gene (NM_006578.3) (Figure 1). Both variants were confirmed by Sanger Sequencing and have already been identified in IDDCA affected individuals. Eight p.(Ser81Leu) homozygous patients, from three different families, have been already described (Lodder et al., 2016; Shamseldin et al., 2016). The pathogenicity of this allele is supported by *in silico* modeling and functional studies. The replacement of the evolutionary conserved Serine 81 with hydrophobic Leucine, buried into the first WD40 protein domain (WD1), would abolish hydrogen bond formation with V108 and bulkier side chain of Leucine at this position would not fit into the tightly packed antiparallel β-sheet of WD1 (Shamseldin et al., 2016). Thus, S81L substitution is predicted to induce localized structural changes that might compromise protein folding and/or stability as well as impair the binding kinetics of RGS proteins (Lodder et al., 2016), and its capacity to deactivate G-protein signaling initiated by dopamine receptors (Shamseldin et al., 2016).

The c.222_226delTAAGA; p.(Asp74Glufs^∗^52) variant, absent in ExAC, has been reported as causative of IDDCA in a recent study (Vernon et al., 2018). This variant is predicted to be loss of function (lof) because losing these nucleotides creates a premature stop codon of the protein after 50 amino acid residues, which is positioned in the second WD-40 domain (Figure 1).

## Discussion

The GNB proteins are expressed in different and specific tissues and interact with many effectors to elicit a wide range of specific cellular responses. Therefore it is not surprising for variants that alter G-proteins functions to compromise the appropriate cellular response, which is associated with aberration of physiological functions, and thus linked with a predisposition to develop human diseases. In humans, variants in each of the five distinct G proteins β-subunits (*GNB1*-*5*) cause rare genetics diseases.

Here we described the second case of a patient who is a compound heterozygote for a null and a missense variant in *GNB5*. Although the number of identified *GNB5* pathogenic variants is still small, there is a hot spot for *GNB5* variants in exon 2 (55% of identified pathogenetic variants), encoding the first WD40 protein domain of the seven that characterize *GNB5* (Figures 1B,C). The WD40 domain shows a β-propeller structure that provides a stable scaffold for protein-protein interaction and is involved in a variety of cellular functions including signal transduction autophagy, and apoptosis (Li and Roberts, 2001). Variants in WD40 domains are associated with an increasing number of human diseases, reviewed in (Laskowski et al., 2016). Of relevance, all the *GNB5* variants reported so far affect highly conserved amino acid residues and WD40 domains that may impair the correct GNB5 protein folding, compromising its function and then the cellular downstream processes leading to the clinical phenotype seen in the patients.

Although more patients are needed to definitively establish a genotype-phenotype correlation, some preliminary speculation has been made. For instance, most patients with missense variants, commonly the p.(Ser81Leu) variant, have mild ID and do not have epileptic encephalopathy (Table 1). In contrast, the majority of patients carrying lof *GNB5* alleles have early infantile developmental and epileptic encephalopathy as well as more significant ID. There is only one other patient in the literature who is compound heterozygous for a missense and lof GNB5 variant (Vernon et al., 2018). Our patient appears to have more mild clinical and neurological manifestations than this previously described patient. Features in common include psychomotor delay and sinus pauses. His psychomotor delay is more significant, however, since he is nonverbal and unable to sit independently at 17 months. He is also described as having laryngomalacia, hearing loss, and a reduction in cone and rod function; none of these phenotypes are present in our patient.

**Table 1:** Clinical features of GNB5-patients reported so far.

	Lodder et al., 2016 Family A II.1	Lodder et al., 2016 Family A II.2	Lodder et al., 2016 Family B II.1	Lodder et al., 2016 Family C II.2	Lodder et al., 2016 Family C II.3	Lodder et al., 2016 Family D II.2	Lodder et al., 2016 Family E II.1	Lodder et al., 2016 Family E II.2	Lodder et al., 2016 Family F II.1
Paternal allele	c.249G > A p.Asp84Valfs^∗^52	c.249G > A p.Asp84Valfs^∗^52	c.249+1G > T p.Asp84Leufs^∗^31	c.249+3A > G p.Asp84Valfs^∗^31	c.249+3A > G p.Asp84Valfs^∗^31	c.906C > G p.Tyr302^∗^	c.242C > T p.Ser81Leu	c.242C > T p.Ser81Leu	c.242C > T p.Ser81Leu
Maternal allele	c.994C > T p.Arg332^∗^	c.994C > T p.Arg332^∗^	c.249+1G > T p.Asp84Leufs^∗^31	c.249+3A > G p.Asp84Valfs^∗^31	c.249+3A > G p.Asp84Valfs^∗^31	c.906C > G p.Tyr302^∗^	c.242C > T p.Ser81Leu	c.242C > T p.Ser81Leu	c.242C > T p.Ser81Leu
Gender, age (years)	F, 24	F, 22	F, 8	F, 13	M, 11	F, 14	F, 15	M, 10	M, 25
Birth weight (percentile)	3,580 g (50th)	NA	2,980 g (15th)	2,751 g (15th)	NA	2,845 g (15th)	NA	NA	NA
Ethnicity	Italy	Italy	Jordan	Puerto Rico	Puerto Rico	India	Morocco	Morocco	Brazil
Consanguinity	-	-	+	+	+	-	-	-	+
Verbal understanding	NA	NA	nonverbal	unremarkable	unremarkable	NA	NA	NA	NA
Speech Development	+	+	nonverbal	nonverbal	delayed	nonverbal	+	+	NA
Intellectual disability (ID)	Severe ID	Severe ID	Severe ID	Severe ID	Global developmental delay	Severe ID	Mild ID	Mild ID	Mild ID
Epilepsy	+	+	+	-	-	+	-	-	-
Retinal Disease	NA	Retinal degeneration	NA	NA	NA	NA	NA	NA	Keratoconus
Nystagmus	+	+	+	+	+	+	NA	-	NA
Sinus sick syndrome	+	Bradyarrhythmia	+	+	+	increased PR interval/	+	+	+
						(intermittent Wenckebach)			
Minimum heart rate (bpm)	24	39	NA	paced	paced	NA	20	16	
Maximum heart rate (bpm)	163	192	NA	paced (27% heartbeats on Holter)	paced (20% heartbeats on Holter)	NA	176	180	NA
Chronotropic response	NA	NA	NA	+	+	NA	unremarkable	unremarkable	NA
Escape beats	+	+	NA	paced	paced	NA	+	+	NA
Pacemaker implantation	-	-	-	+	+	-	-	+	NA
Cardiac anomalies	-	PFO	NA	-	-	-	-	-	NA
Hypotonia	+	+	+	+	+	+	-	impaired fine motor skills	-
Gastroesophageal Reflux	+	+	-	+	+	+	-	-	NA
Others				Abnormally mitochondrial shape, focal z-band streaming and type1 fiber predominance					
Dysmorphic feature(s)									
Paternal allele	c.242C > T p.Ser81Leu	c.242C > T p.Ser81Leu	c.242C > T p.Ser81Leu	c.242C > T p.Ser81Leu	c.242C > T p.Ser81Leu	c.355delG p.Ala119Profs^∗^16	c.355delG p.Ala119Profs^∗^16	c.737G > A p.Arg246Gln	c.222_226delTAAGA p.Asp74Glufs^∗^52
Maternal allele	c.242C > T p.Ser81Leu	c.242C > T p.Ser81Leu	c.242C > T p.Ser81Leu	c.242C > T p.Ser81Leu	c.242C > T p.Ser81Leu	c.355delG p.Ala119Profs^∗^16	c.355delG p.Ala119Profs^∗^16	c.222_226delTAAGA p.Asp74Glufs^∗^52	c.242C > T p.Ser81Leu
Gender, age (years)	F, 12	F, 11	F, 5	F, 7	F, 11	M, 3	F, 11	M, 2	F, 2.5
Birth weight (percentile)	50th centile	50th centile	NA	50th centile	NA	NA	1800 g ( < 1st)	3311 g (50th)	1698 g ( < 1st) (Intrauterine growth restricted)
Ethnicity	Saudi	Saudi	Saudi	Saudi	Saudi	NA	NA	European/Caucasian	European/Caucasian
Consanguinity	+	+	+	-	-	+	+	-	-
Verbal understanding	+	+	+	+	+	“no developmental milestones”	“no developmental milestones”	nonverbal	+
Speech Development	Severe language delay	Severe language delay	Severe language delay	Severe language delay	Severe language delay	“no developmental milestones”	“no developmental milestones”	nonverbal	expressive speech delay
Intellectual disability (ID)	Normal IQ, but school performance issues	Normal cognitive development	NA	Normal IQ	NA	Severe ID	Severe ID	Severe ID	Mild ID
Epilepsy	NA	NA	NA	NA	NA	+	+	-	-
Retinal Disease	NA	NA	NA	NA	NA	Retinal degeneration	Retinal degeneration	Severe reduction in cone and rode function	-
Nystagmus	NA	NA	NA	NA	NA	+	+	+	Strabismus
Sinus sick syndrome	NA	NA	NA	NA	NA	Sinus arrhythmia/ Sinus bradycardia	Sinus arrhythmia/ Sinus bradycardia	Sinus arrhythmia/ Sinus bradycardia	+
Minimum heart rate (bpm)	NA	NA	NA	NA	NA	NA	NA	71	36
Maximum heart rate (bpm)	NA	NA	NA	NA	NA	NA	NA	183	176
Chronotropic response	NA	NA	NA	NA	NA	NA	NA	NA	NA
Escape beats	NA	NA	NA	NA	NA	NA	NA	+ (prior to pacing)	+
Pacemaker implantation	NA	NA	NA	NA	NA	Refused by parents	Refused by parents	+	+
Cardiac anomalies	NA	NA	NA	NA	NA	-	-	-	-
Hypotonia	-	-	NA	+	NA	+	+	+	+
Gastroesophageal Reflux	NA	NA	NA	NA	NA	normal (abdominal US examination)	normal (abdominal US examination)	+	-
Others	ADHD, Hyperactivity	Inattentive type ADHD	Motor delay	Motor delay	ADHD, mild motor delay	autistic (midline hand automatism, no eye contact)	autistic (midline hand automatism, no eye contact)	center-sided hearing loss, hypertonia, upper extremity jerking motions, laryngomalacia hypertonia	tympanostomy tubes
Dysmorphic feature(s)						prominent forehead, micro-brachycephaly (acquired)	prominent forehead, micro-brachycephaly (acquired)	thin corpus callosum (brain MRI)	


*M, male: F, female; NA, not available; +, clinical trait present; -, clinical trait not present; bpm, beats per minute; ADHD, attention deficit hyperactivity disorder; PFO, patent foramen ovale; US, ultrasound.*

These two patients have the same p.(Asp74Glufs^∗^52) lof variant; however, our newly reported patient has the commonly reported p.(Ser81Leu) missense variant whereas the patient described in Vernon et al. (2018) has a novel c.737G > A variant. The latter might affect splicing and, although experimental assays are needed, it could cause a lof, a type of variant more consistent with the phenotype seen in that patient.

This study corroborates the direct correlation between the type of the *GNB5* genetic variant and the severity of related phenotype. Individuals with missense variants, both in homozygous or compound heterozygous states, present with a less severe/moderate phenotype characterized mainly by sinus node dysfunction in combination with mild intellectual disabilities; whereas individuals homozygous for null alleles have severe ID, global developmental delay including early infantile developmental and epileptic encephalopathy, hypotonia, as well as sinus node dysfunction (Lodder et al., 2016).

## Concluding Remarks

This report extends the number of individuals carrying GNB5 pathogenic variant, and demonstrates the phenotypic consequences of heterotrimeric G-proteins deficiency and their critical roles in neuronal and cardiac manifestations. Additional patient studies will help us to more consistently predict phenotype based on their molecular results. Similarly, the combination of functional studies and cellular modeling of the disease (e.g., iPSC-derived heart and brain lineage cells) will provide new insights on the pathogenesis of the disease.

## Author Contributions

GM conceived and designed the study, and provided the funding and supervision. ST, WW, and KK identified the patient and collected clinical follow-up data. GM, GMS, and NM revised the literature and collected data. NM, ST, and GM wrote the manuscript. All authors edited the manuscript.

## Conflict of Interest Statement

GM is a paid consultant for Takeda Pharmaceutical Company. The remaining authors declare that the research was conducted in the absence of any commercial or financial relationships that could be construed as a potential conflict of interest.

## Footnotes

1 https://www.genedx.com/
